# The Relationship Between Weekend Catch‐Up Sleep and Hypertension: Insights From the National Health and Nutrition Examination Survey, 2017–2023

**DOI:** 10.1155/ijhy/1755790

**Published:** 2026-08-30

**Authors:** Han Chen, Qiang Tong, Lu Chai, Yongqi Shan

**Affiliations:** ^1^ Department of Pharmacy, General Hospital of Northern Theater Command (General Hospital of Shenyang Military Command), Shenyang 110016, Liaoning, China; ^2^ Department of General Surgery, General Hospital of Northern Theater Command (General Hospital of Shenyang Military Command), Shenyang 110016, Liaoning, China

**Keywords:** American adults, hypertension, subgroup analysis, weekend catch-up sleep

## Abstract

**Objective:**

Sleep deprivation is associated with an increased risk of hypertension (HTN), and many individuals practice weekend catch‐up sleep (WCS). However, evidence of an association between WCS and HTN in adults in the United States remains limited. This study examines this relationship using a nationally representative sample.

**Methods:**

Data from NHANES 2017–2023 (16,268 adults aged ≥ 20 years) were analyzed. The WCS was defined as weekend sleep minus weekday sleep. Multivariate logistic regression was used to adjust for sociodemographic, lifestyle, and metabolic factors. WCS was examined as a binary (> 0 vs. ≤ 0 h), continuous, and categorical (≤ 0, 0–1, 1–2, 2–3, and ≥ 3 h) variable. Subgroup analyses were exploratory and not corrected for multiple testing.

**Results:**

In the fully adjusted models, neither the binary nor continuous WCS duration was significantly associated with HTN (*p* > 0.05). Among categorical analyses, only the 0–1 h WCS group showed lower odds of HTN compared with the ≤ 0 h group (OR = 0.814, 95% CI: 0.666–0.995, *p* = 0.033); all other WCS categories (1–2 h, 2–3 h, and ≥ 3 h) were nonsignificant. No dose–response relationship was observed. Subgroup analyses suggested potential effect modification by sex and education, but these results are hypothesis‐generating given the lack of correction for multiple comparisons.

**Conclusion:**

In this large cross‐sectional study, the WCS was not robustly associated with HTN. The isolated finding of 0–1 h of WCS requires confirmation in prospective studies and should not be interpreted as evidence for a preventive or therapeutic strategy.

## 1. Introduction

Insufficient sleep is a significant issue that affects individuals in contemporary society. The National Sleep Foundation advises that adolescents should obtain at least 8 h of sleep, while adults should aim for at least 7 h to sustain optimal health and well‐being [1]. However, fulfilling these recommendations is challenging due to academic and occupational commitments. Consequently, many individuals increasingly compensate for sleep deficits by extending their sleep duration on weekends, a practice commonly referred to as weekend catch‐up sleep (WCS).

Cardiovascular diseases (CVDs) are a spectrum of disorders affecting the cardiovascular system [2]. CVDs remain the foremost cause of death globally, accounting for almost 17.9 million deaths annually. Beyond the substantial loss of human life, CVDs impose a considerable economic burden, with costs exceeding $400 billion annually. This financial impact is anticipated to escalate to $1.49 trillion by 2050 in the United States alone [3]. Hypertension (HTN) is a prevalent condition among individuals with CVDs, affecting 31.1% of the adult population [4, 5]. It is an independent risk factor for CVDs; consequently, its effective management is crucial in CVD treatment approaches [6].

Despite the extensive body of research, most prior investigations have primarily concentrated on the correlation between HTN and sleep duration. Scott et al. demonstrated a correlation between sleep irregularity and HTN in a large global population sample [1]. Similarly, Luo et al. emphasized that inadequate weekday sleep duration substantially increased the risk of HTN among adolescents [7]. However, the effects of WCS on HTN in adults remain unclear. Given this gap in the literature, a nationally representative study of the American population could provide critical insights into this issue, thereby contributing to the prevention and management of HTN in the United States.

Herein, we sought to evaluate the link between WCS and HTN among adults participating in the National Health and Nutrition Examination Survey (NHANES), with the goal of describing associations rather than informing clinical guidelines. These findings may help generate hypotheses for future prospective studies.

## 2. Methods

### 2.1. Study Population

**FIGURE 1:**
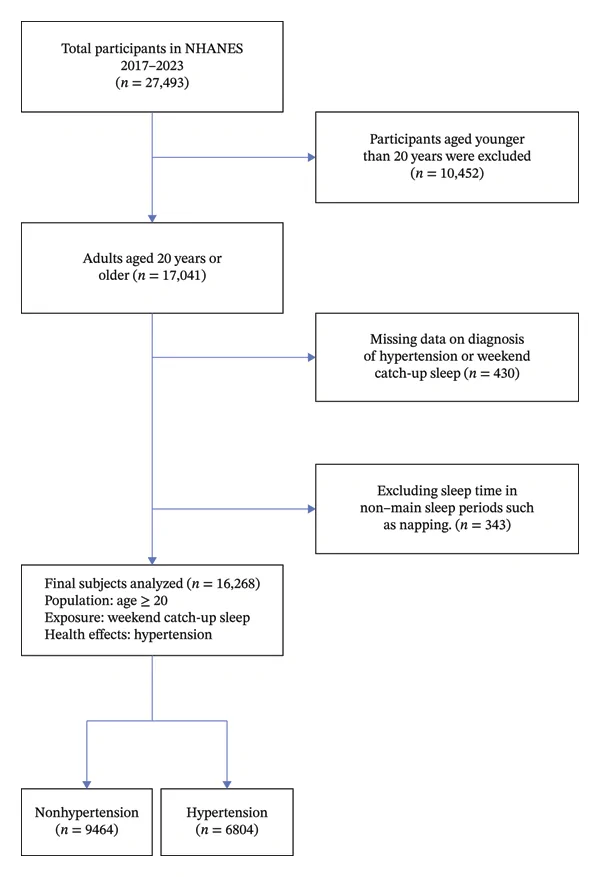
Flowchart of participant selection. NHANES 2017–2023, adults aged ≥ 20 years. Exclusions: age < 20 years, missing blood pressure or sleep data, and missing covariates. Final analytic sample *N* = 16,268.

The NHANES, administered by the Centers for Disease Control and Prevention (CDC), collects cross‐sectional data on the nutrition and health of US children and adults. Since 1999, the NHANES has been conducted biennially using nationally representative samples. Its primary purpose is to estimate the dietary status and health of the US population. Ethical approval was obtained from the National Center for Health Statistics (NCHS) Institutional Review Board, and informed consent was obtained from each participant (https://www.cdc.gov/nchs/nhanes/index.htm). This study analyzed two NHANES cycles (2017–2020 and 2021–2023), including 27,493 participants. The exclusion criteria were as follows: (1) age < 20 years, (2) missing data on HTN diagnosis or WCS, and (3) sleep duration recorded only for nonprimary sleep periods (e.g., naps). Figure 1 illustrates the screening process.

### 2.2. HTN and WCS Assessments

In the NHANES, blood pressure was assessed as the mean of three consecutive systolic blood pressure (SBP) and diastolic blood pressure (DBP) measurements, each taken 60 s apart. HTN was defined according to the 2017 ACC/AHA guideline as follows: (1) mean SBP ≥ 130 mmHg and/or mean DBP ≥ 80 mmHg (based on up to three measurements); (2) self‐report of current use of antihypertensive medication; or (3) self‐report of a prior physician diagnosis of HTN. Participants who met any of these criteria were classified as having HTN [8]. In the 2017–2023 surveys, participants reported sleep duration on (1) weekdays/workdays and (2) weekends/nonworkdays. WCS was defined as the difference between weekend and weekday sleep duration, calculated only for primary sleep periods (excluding naps). WCS was categorized into five groups: no increase (≤ 0 h), very short (> 0 to < 1 h), short (≥ 1 to < 2 h), moderate (≥ 2 to < 3 h), and long (≥ 3 h). These cutoff values were chosen based on previous studies on WCS and cardiovascular outcomes, e.g., Han et al. [9, 10], to facilitate comparability across studies. The mean sleep duration was determined using the following weighted average formula: (5 × weekday sleep duration + 2 × weekend sleep duration)/7 [9]. Numerous studies on sleep duration rely on self‐reported data from participants, which, although subject to some degree of inaccuracy, provide valuable insights [11]. This study utilized self‐reported sleep data from participants.

### 2.3. Covariates

Based on prior literature and clinical relevance, the following covariates were included: sex (male, female), age (≤ 35, 36–64, ≥ 65), and education (below high school, high school, above high school). Ethnicity was categorized as Mexican American, other Hispanic, non‐Hispanic White, or other. Smoking status was defined as never, former, or current, and marital status as separated/divorced/widowed, married/living with a partner, or never married. Body mass index (BMI) was classified as ≤ 25, 25–30, or > 30 kg/m^2^ [11]. Alcohol intake was categorized as nondrinkers (< 12 drinks lifetime), moderate (≥ 1 drink/day for women or ≥ 2 for men in the past year), or heavy (≥ 2 drinks/day for women or ≥ 3 for men) [12]. The poverty–income ratio (PIR) was grouped as < 1.3, 1.3–3.5, or ≥ 3.5 [13]. Sedentary behavior was assessed using PAD680, with daily sedentary time > 7.5 h defined as severe and ≤ 7.5 h as mild [14]. Diabetes was identified as a self‐reported diagnosis, HbA1c ≥ 6.5%, or fasting blood glucose ≥ 126 mg/dL (7.0 mmol/L) [2]. Prediabetes was identified as a self‐reported diagnosis, HbA1c 5.7%–6.4%, or fasting blood glucose 5.6–6.9 mmol/L [15].

### 2.4. Statistical Analyses

This study utilized Stata 17 and EmpowerStats (Version 2.0, https://www.empowerstats.com) for data analysis to evaluate the association between WCS and HTN. Descriptive statistics accounting for the survey design were used, with categorical variables reported as frequencies and weighted percentages and continuous variables as means with standard errors (SEs). For the combined analysis of the 2017–2020 and 2021–2023 cycles, we followed the NCHS recommendations for pooling multiple 2‐year cycles. Because both cycles have the same 2‐year sampling duration and design, we used the original 2‐year MEC examination weights provided for each cycle (WTMECPRP for 2017–2020 and WTMEC2YR for 2021–2023) without further rescaling, which effectively gives each cycle approximately equal contribution to the pooled estimates. This decision is supported by the NCHS analytic guidelines for combining survey cycles, which recommend using the original weights without rescaling when pooling cycles of equal length and similar design [16]. All regression models accounted for the complex survey design using the complex survey module in EmpowerStats (Version 2.0), with the weighting, stratification (SDMVSTRA), and clustering (SDMVPSU) variables specified accordingly. Baseline differences between the WCS and non‐WCS groups were tested using weighted chi‐square tests for categorical variables and weighted linear regression for continuous variables. The association between WCS and HTN was examined using multiple logistic regression analysis. WCS was the primary independent variable, defined as > 0 h (yes/no), while WCS duration served as a secondary variable, analyzed continuously and categorically (≤ 0, 0–1, 1–2, 2–3, and ≥ 3 h) [17]. Three models were constructed: Model 1 was unadjusted; Model 2 was adjusted for age, sex, and ethnicity; and Model 3 was adjusted for all covariates (age, sex, ethnicity, education, marital status, PIR, alcohol intake, smoking, BMI, sedentary behavior, diabetes, prediabetes, HTN, average sleep, and weekday sleep). We acknowledge that WCS is derived from weekday and weekend sleep and that including average sleep duration and weekday sleep duration in Model 3 may introduce collinearity. However, we included them to assess whether WCS had an effect independent of absolute sleep time. Collinearity diagnostics (variance inflation factors [VIFs]) were performed, and all VIF values were < 3 (Table 1), indicating no severe multicollinearity. In the sensitivity analysis excluding average sleep duration and weekday sleep duration from Model 3, the estimates remained largely unchanged. Compared with the ≤ 0 h reference group, the 0–1 h category showed an OR of 0.81 (95% CI: 0.67–0.98, *p* = 0.031); the 1–2 h category showed an OR of 1.02 (95% CI: 0.88–1.19, *p* = 0.78); the 2–3 h category showed an OR of 0.95 (95% CI: 0.78–1.16, *p* = 0.61); and the ≥ 3 h category showed an OR of 0.97 (95% CI: 0.79–1.20, *p* = 0.75). These results indicate that the inclusion of average sleep and weekday sleep in the fully adjusted model did not materially alter the findings. Stratified analyses were conducted to assess the associations across subgroups and potential interactions.

**TABLE 1:** Demographic and clinical characteristics of adults with and without weekend catch‐up sleep in the NHANES 2017–2023 cycles (*n* = 16,268).

Variables	Total (*N* = 16,268)	Non‐WCS (*N* = 9236)	WCS (*N* = 7032)	*p* value
Age (years)	48.7 ± 0.4	52.8 ± 0.5	43.9 ± 0.4	< 0.001
≤ 35	28.7 (26.9–30.5)	23.6 (21.8–25.6)	34.5 (32.5–36.4)	
36–64	49.5 (48.0–51.0)	43.7 (41.8–45.6)	56.2 (54.2–58.2)	
≥ 65	21.8 (20.1–23.7)	32.7 (30.3–35.2)	9.4 (8.3–10.6)	
Gender (*N*, weighted %)				0.133
Men	48.0 (46.9–49.2)	48.6 (47.2–49.9)	47.4 (45.7–49.1)	
Women	52.0 (50.8–53.1)	51.4 (50.1–52.8)	52.6 (50.9–54.3)	
Race/ethnicity (*N*, weighted %)				< 0.001
Mexican American	8.0 (6.1–10.4)	6.2 (4.6–8.3)	10.1 (7.7–12.9)	
Other Hispanic	8.3 (7.1–9.8)	7.2 (5.8–8.7)	9.7 (8.4–11.2)	
Non‐Hispanic White	62.0 (58.6–65.4)	65.5 (61.6–69.3)	58.0 (54.7–61.3)	
Non‐Hispanic Black	10.9 (9.0–13.1)	10.7 (8.7–13.2)	11.0 (9.2–13.1)	
Other races	10.8 (9.3–12.5)	10.4 (8.8–12.2)	11.3 (9.7–13)	
Education level (*N*, weighted %)				< 0.001
Less than high school	10.7 (9.7–11.7)	11.3 (10.4–12.3)	9.9 (8.6–11.5)	
High school	26.3 (24.2–28.5)	28.2 (26–30.4)	24.2 (21.7–27)	
More than high school	62.9 (60.2–65.6)	60.5 (57.8–63.1)	65.8 (62.3–69.1)	
Not recorded	0.0 (0.0–0.1)	0.1 (0.0–0.1)	0.0 (0.0–0.1)	
Marital status (*N*, weighted %)				< 0.001
Married/living with partner	61.9 (60.0–63.8)	60.2 (58.0–62.2)	63.9 (61.6–66.1)	
Widowed/divorced/separated	18.6 (17.5–19.7)	21.7 (20.3–23.2)	15.0 (13.8–16.3)	
Never married	19.5 (18–21.1)	18.1 (16.4–20)	21.1 (19.3–22.9)	
Not recorded	0.0 (0.0–0.1)	0.0 (0.0–0.1)	0.0 (0.0–0.1)	
Poverty–income ratio (*N*, weighted %)				< 0.001
< 1.3	16.8 (15.3–18.3)	18.5 (16.8–20.3)	14.8 (13.2–16.5)	
1.3–3.5	31.5 (29.3–33.8)	32.2 (29.3–35.3)	30.7 (28.6–32.8)	
> 3.5	39.8 (37.3–42.2)	36.3 (33.4–39.3)	43.7 (40.9–46.5)	
Not recorded	12 (10.8–13.3)	13 (11.3–14.8)	10.8 (9.7–12.0)	
Alcohol drinking status (*N*, weighted %)				< 0.001
Nondrinkers	6.8 (6.1–7.5)	7.4 (6.5–8.4)	6.1 (5.3–6.9)	
Moderate drinkers	34.9 (33.2–36.7)	35 (33.2–36.9)	34.8 (32.7–36.9)	
Heavy drinkers	39.8 (38.1–41.6)	36.4 (34.7–38.2)	43.8 (41.5–46.1)	
Not recorded	18.5 (17.2–19.8)	21.2 (19.6–22.9)	15.3 (13.9–16.9)	
Smoking status (*N*, weighted %)				< 0.001
Never	59.5 (57.6–61.4)	56.1 (53.6–58.6)	63.4 (61.3–65.4)	
Former	25.1 (23.7–26.5)	27.3 (25.5–29.1)	22.5 (20.8–24.4)	
Now	15.4 (13.9–16.9)	16.5 (15–18.2)	14 (12.2–16.1)	
Not recorded	0.1 (0–0.1)	0.1 (0.0–0.2)	0.0 (0.0–0.1)	
BMI (*N*, weighted %)				< 0.001
< =25 kg/m^2^	27.2 (25.6–28.9)	27.1 (25.4–28.9)	27.3 (25.3–29.5)	
25–30 kg/m^2^	30.8 (29.7–32)	31.9 (30.5–33.3)	29.6 (27.9–31.4)	
> 30 kg/m^2^	40.7 (38.6–42.7)	39.4 (37.5–41.3)	42.2 (39.3–45.1)	
Not recorded	1.3 (1–1.5)	1.6 (1.3–1.9)	0.9 (0.6–1.2)	
Sedentary behavior (*N*, weighted %)				0.005
≤ 7.5 h	66.9 (65.3–68.5)	67.9 (66.4–69.4)	65.8 (63.2–68.3)	
> 7.5 h	32.5 (31–34.1)	31.5 (30–33)	33.7 (31.2–36.3)	
Not recorded	0.6 (0.4–0.7)	0.6 (0.4–0.9)	0.5 (0.3–0.8)	
Antihypertensive medication use, (*N*, weighted %)				< 0.001
No		13.9 (11.2–14.5)	16.6 (13.5–18.2)	
Yes		86.1 (82.4–88.5)	83.4 (80.1–84.3)	
Diabetes (*N*, weighted %)				< 0.001
No	88.2 (87.4–89)	86.4 (85.4–87.4)	90.3 (89–91.5)	
Yes	11.8 (11–12.6)	13.6 (12.6–14.6)	9.7 (8.5–11.0)	
Prediabetes (*N*, weighted %)				< 0.001
No	66.1 (64.7–67.4)	63.9 (62.4–65.4)	68.6 (66.5–70.5)	
Yes	33.9 (32.5–35.2)	36 (34.6–37.5)	31.4 (29.4–33.4)	
Not recorded	0.1 (0–0.1)	0.1 (0.0–0.2)	0.1 (0.0–0.2)	
Hypertension (*N*, weighted %)				< 0.001
No	62.3 (60.7–63.8)	58.9 (56.8–61.1)	66.1 (63.9–68.2)	
Yes	37.7 (36.2–39.3)	41.1 (38.9–43.2)	33.9 (31.8–36.1)	
Average sleep duration, mean (SE)	7.8 ± 0.02	7.9 ± 0.02	7.7 ± 0.02	< 0.001
Weekday sleep duration, mean (SE)	7.6 ± 0.02	8.0 ± 0.02	7.2 ± 0.02	< 0.001

## 3. Results

### 3.1. Characteristics of Participants

In a sample of 16,268 adults, 7032 were categorized into the WCS group, representing a weighted proportion of 43.2%. The study found that participants in the WCS group exhibited significantly shorter weekday sleep durations and average sleep durations than those in the non‐WCS group (*p* < 0.001). Additionally, the distribution of WCS was significantly associated with HTN status (*p* < 0.001). Furthermore, the WCS group demonstrated statistically significant variations in most sociodemographic and health‐related variables when compared with the non‐WCS group, with the exception of sex (Table 1).

### 3.2. Relationship Between WCS and HTN

In the primary analysis, the association between WCS and HTN was evaluated using multivariate logistic regression (Table 2). WCS was analyzed as a binary (yes/no), continuous, and categorical variable (≤ 0, 0–1, 1–2, 2–3, and ≥ 3 h). Although both WCS and continuous WCS durations were negatively associated with HTN, these associations were not significant (all *p* > 0.05). Compared with individuals with ≤ 0 h WCS, those with 0–1 h WCS had lower odds of HTN, with ORs of 0.623 (*p* < 0.001), 0.761 (*p* = 0.005), and 0.814 (*p* = 0.033) in Models 1, 2, and 3, respectively. Because multiple comparisons were performed (e.g., five WCS categories and numerous subgroup analyses), the single significant finding for the 0–1 h category in Model 3 may be a chance finding. No correction for multiple testing (e.g., Bonferroni) was applied; therefore, this result should be interpreted as exploratory.

**TABLE 2:** Logistic regression analyses for associations between WCS and hypertension.

Variables	Model 1		Model 2		Model 3	
	OR (95% CI)	*p* value	OR (95% CI)	*p* value	OR (95% CI)	*p* value
WCS (duration > 0 h)						
No	Ref.		Ref.		Ref.	
Yes	0.736 (0.653–0.831)	0	0.924 (0.813–1.049)	0.213	0.908 (0.761–1.084)	0.279
WCS duration (continuous)	0.946 (0.912–0.981)	0.004	0.995 (0.958–1.033)	0.806	0.995 (0.958–1.033)	0.789
WCS duration (multicategory)						
≤ 0 h	Ref.		Ref.		Ref.	
0∼1 h	0.623 (0.519–0.748)	0	0.761 (0.631–0.917)	0.005	0.814 (0.674–0.983)	0.033
1∼2 h	0.788 (0.683–0.908)	0.002	0.975 (0.841–1.13)	0.729	0.978 (0.801–1.193)	0.82
2∼3 h	0.716 (0.582–0.881)	0.002	0.914 (0.746–1.12)	0.377	0.885 (0.628–1.247)	0.476
≥ 3 h	0.774 (0.631–0.949)	0.015	1.022 (0.819–1.274)	0.845	0.907 (0.607–1.355)	0.626

### 3.3. Subgroup Analyses

Subgroup analyses stratified by sex, age, education, and other covariates were performed as exploratory analyses without adjustment for multiple comparisons. Because these analyses were not prespecified and multiplicity was not controlled, they should be interpreted as hypothesis‐generating only. The complete results for all WCS categories across all subgroups are provided in Supporting Table 1. In the main text, we report only the interaction *p* values: Significant interactions were observed for sex (*p*_interaction_ = 0.029) and education (*p*_interaction_ = 0.027); no other interaction reached statistical significance. No separate figure is presented for subgroup results, given their exploratory nature and the largely null primary findings.

## 4. Discussion

### 4.1. Main Outcomes

There is limited evidence regarding the impact of WCS on HTN in adults. To the best of our knowledge, this is the first study to examine this association in American adults. Weekday and weekend sleep durations were obtained using a questionnaire. In this nationally representative cohort, no significant association was observed between WCS and HTN after adjusting for all covariates. Notably, individuals who engaged in WCS for 0–1 h, compared with those with no increase in sleep duration (WCS ≤ 0 h), had a lower prevalence of HTN. Although some subgroup analyses suggested potential effect modification by sex and education (Supporting Table 1), these results emerged from unplanned comparisons without multiplicity correction. The interaction *p* values were borderline (*p* = 0.029 and 0.027, respectively) and were not consistent across WCS categories. For instance, the apparent protective association of 0–1 h WCS was observed only in certain subgroups (e.g., women, heavy drinkers, and nondiabetic individuals) but not in others, with no clear pattern across the strata. Given the largely null primary findings and the exploratory nature of these analyses, these subgroup results should not be overinterpreted and do not support differential clinical recommendations. They are presented solely to generate hypotheses for future prospective studies. We also acknowledge that adjusting for variables mathematically related to the exposure (e.g., average sleep duration and weekday sleep duration) remains a methodological decision rather than a universally accepted approach in epidemiological modeling; our sensitivity analyses without these variables yielded similar results, suggesting that our main findings are robust to this modeling choice.

#### 4.1.1. Epidemiological and Experimental Evidence

Earlier studies have investigated the relationship between sleep duration and HTN. Studies have demonstrated that insufficient sleep on weekdays is associated with an increased risk of HTN among adolescents [17]. In a cohort study involving 430 American adolescents, shorter weekday sleep duration (< 8 h) was significantly associated with higher odds of HTN (OR, 1.092; 95% CI, 1.042–1.144), whereas prolonged sleep during weekends did not confer a protective effect. In contrast, a longer total weekly sleep duration (greater than 10 h) was associated with a lower risk of HTN, whereas discrepancies between weekday and weekend sleep durations were not significant factors. These findings differ slightly from ours, which indicate that WCS is not a significant factor associated with HTN in American adults. This discrepancy may be attributed to population heterogeneity and the differing physiological features between adolescents and adults. There remains a paucity of research exploring why sleep impacts blood pressure differently in adolescents than in adults.

The mechanisms linking insufficient sleep to HTN remain incompletely understood; previous studies have suggested involvement of autonomic dysfunction, inflammation, endothelial impairment, and circadian rhythm disruption. However, because the present study found no consistent association across the WCS categories and no dose–response relationship, our data do not provide mechanistic evidence. The mechanisms discussed above were derived from other studies on sleep deprivation and should not be inferred from our results. We therefore keep this section brief and refrain from extensive mechanistic speculation. Specifically, previous studies have shown that sleep deprivation elevates sympathetic function while suppressing parasympathetic tone, resulting in elevated blood pressure. Notably, studies indicate that 24‐h sleep deprivation elevates blood pressure in young men and SBP in older adults, particularly women [18]. Our results do not provide direct evidence for sex‐specific effects; the observed subgroup differences are exploratory and require confirmation.

However, because our primary analyses did not show a consistent or dose‐dependent association between WCS and HTN, we could not draw strong conclusions regarding the potential benefits of the weekend sleep extension. Although some previous studies (e.g., in Korean adults [10]) have reported that compensatory weekend sleep might reduce the risk of HTN, our results do not robustly support such an effect. Mechanistic interpretations derived from our data remain speculative. Future research should further investigate these biological mechanisms using objective sleep measurements and prospective designs.

#### 4.1.2. Implications and Recommendations

This study evaluated the association between WCS and HTN in a nationally representative cohort. Given the mostly null findings and lack of a dose–response relationship, this study does not support the use of WCS as a strategy for HTN prevention or management. Future studies should use prospective designs and objective sleep measurements.

#### 4.1.3. Strengths and Limitations

Our study had several notable strengths. First, the participants were sourced from a large, nationally representative survey (NHANES 2017–2023), which ensures the reliability and generalizability of our findings. Second, we utilized the most recent NHANES data from 2021–2023 and excluded nonprimary sleep periods, such as napping, from our analysis. Napping may influence the prevalence of HTN. Yang et al. [19] identified an elevated daytime nap frequency as a potential causal risk factor for essential HTN [18]. Recent investigations have shown that midday napping may be a risk factor for HTN, with BMI serving as a mediator [20]. Therefore, when examining the effects of WCS, it is important to exclude midday naps and other non–catch‐up sleep periods to facilitate an accurate assessment. Third, the analytical methods employed in this study were comprehensive and robust. Specifically, survey‐weighted logistic regression, subgroup, and interaction analyses were conducted to thoroughly investigate the association between WCS and HTN. However, this study had numerous limitations. The primary limitation was the inability to establish a causal association between WCS and HTN owing to the cross‐sectional design of the study. Despite this, the results suggest promising directions for future longitudinal investigations to evaluate causal relationships. Furthermore, the WCS assessment depended exclusively on self‐reported questionnaires, which introduced the potential for recall bias. Future research should aim to incorporate more reliable and precise sleep data using objective measurement techniques to validate these findings. Additionally, although efforts were made to adjust for confounding variables, there may still be unmeasured factors that could influence the study’s conclusions. Reverse causality is possible; individuals who have been diagnosed with HTN or who are aware of their elevated blood pressure may consciously alter their weekend sleep patterns (e.g., sleep longer or shorter), which could bias the observed associations. The cross‐sectional design precludes the determination of the direction of any relationship; however, despite these limitations, this investigation represents the first large‐scale examination of the association between WCS duration and HTN. Furthermore, the reference category (WCS ≤ 0 h) combined participants with no weekend sleep extension and those with negative WCS (i.e., shorter weekend sleep than weekday sleep). These two groups may be physiologically heterogeneous, as negative WCS could reflect severe sleep debt or irregular sleep patterns. However, the sample size in the negative‐only subgroup was insufficient for a separate analysis in our study. Future studies with larger samples should consider separating negative, zero, and positive WCS to strengthen the biological interpretation.

## 5. Conclusions

In this nationally representative cross‐sectional study of US adults, WCS, as a binary or continuous variable, was not significantly associated with HTN after full adjustment. A single exploratory finding of lower HTN odds in the 0–1 h WCS group was not supported by a dose–response relationship or by consistency across other WCS categories. Given the lack of correction for multiple testing and the largely negative primary analyses, these results should be considered hypothesis‐generating. Prospective studies with objective sleep measures are required to determine whether any form of compensatory weekend sleep is associated with the risk of developing HTN.

**Nomenclature**
BMI: Body mass index
CI: Confidence interval
NHANES: National Health and Nutrition Examination Survey
OR: Odds ratio
SE: Standard error
WCS: Weekend catch‐up sleep

## Author Contributions

Conceptualization: Han Chen and Qiang Tong; methodology, software, resources, data curation, and writing–original draft preparation: Han Chen; validation: Han Chen, Qiang Tong, and Lu Chai; formal analysis and writing–review and editing: Yongqi Shan; investigation: Lu Chai; supervision: Qiang Tong and Lu Chai.

## Funding

This study did not receive any funding.

## Disclosure

All authors have read and agreed to the published version of the manuscript.

## Ethics Statement

The NHANES protocol was reviewed and approved by the National Center for Health Statistics Institutional Review Board, and all participants provided written informed consent. The present study analyzed publicly available, deidentified secondary data; therefore, additional institutional review board approval was not required.

## Conflicts of Interest

The authors declare no conflicts of interest.

## Supporting Information

Additional supporting information can be found online in the Supporting Information section.

## Supporting information


**Supporting Information** Supporting Table 1. Hierarchical analysis and interaction effects.

## Data Availability

Data from the National Health and Nutrition Examination Survey (2017–2020) are publicly available at the NHANES website for the 2017–2020 and 2021–2023 survey cycles. https://wwwn.cdc.gov/nchs/nhanes/continuousnhanes/default.aspx?Cycle=2017-2020 https://wwwn.cdc.gov/nchs/nhanes/continuousnhanes/default.aspx?Cycle=2021-2023. All the data are available upon request.
