# Supplementary material for: The Relationship Between Weekend Catch‐Up Sleep and Hypertension: Insights From the National Health and Nutrition Examination Survey, 2017–2023
Source: Int J Hypertens. 2026 Aug 30;2026:1755790. doi: 10.1155/ijhy/1755790 (PMC13526226; doi:10.1155/ijhy/1755790)
Supplement: Supplementary file 1 [file IJHY-2026-1755790-s001.docx]

**Supplementary Table 1.**Hierarchical analysis and interaction effects.

| Variblesa | Weekend catch-up sleep groups | | | | | | | | | | | | |  | p for interaction |
| --- | --- | --- | --- | --- | --- | --- | --- | --- | --- | --- | --- | --- | --- | --- | --- |
|  | No increase in sleep duration |  | Very short catch-up sleep duration | |  | Short catch-up sleep duration | |  | Moderate catch-up sleep duration | |  | Long catch-up sleep duration | |  |  |
|  | (WCS ≤ 0 h) |  | (0 h < WCS ＜ 1 h) | P value |  | (1 h ≤ WCS < 2 h) | P value |  | (2 h ≤ WCS < 3 h) | P value |  | (WCS ≥ 3 h) | P value |  |  |
| Age (years) |  |  |  |  |  |  |  |  |  |  |  |  |  |  | 0.297 |
| ≤ 35 | ref |  | 0.987  (0.626-1.555) | 0.952 |  | 1.109(0.847-1.453) | 0.443 |  | 1.159(0.776-1.732) | 0.461 |  | 1.054(0.636-1.747) | 0.83 |  |  |
| 36-64 | ref |  | 0.752  (0.588-0.962) | 0.024 |  | 0.998(0.804-1.238) | 0.984 |  | 0.845(0.636-1.122) | 0.237 |  | 0.906(0.678-1.21) | 0.50 |  |  |
| ≥ 65 | ref |  | 1.013  (0.647-1.586) | 0.955 |  | 0.964(0.702-1.325) | 0.819 |  | 0.774(0.488-1.23) | 0.271 |  | 0.925(0.51-1.675) | 0.79 |  |  |
| Gender |  |  |  |  |  |  |  |  |  |  |  |  |  |  | 0.029 |
| Men | ref |  | 0.929  (0.694-1.244) | 0.612 |  | 1.035(0.864-1.241) | 0.700 |  | 0.929(0.715-1.207) | 0.571 |  | 1.079(0.727-1.601) | 0.70 |  |  |
| Women | ref |  | 0.726(0.543-0.971) | 0.032 |  | 0.966(0.77-1.211) | 0.759 |  | 0.89(0.644-1.23) | 0.470 |  | 0.827(0.629-1.089) | 0.17 |  |  |
| Race/ethnicity |  |  |  |  |  |  |  |  |  |  |  |  |  |  | 0.890 |
| Mexican American | ref |  | 0.681  (0.399-1.162) | 0.154 |  | 0.921(0.566-1.5) | 0.735 |  | 1.011(0.734-1.392) | 0.947 |  | 0.728(0.46-1.152) | 0.17 |  |  |
| Other Hispanic | ref |  | 0.755(0.438-1.3) | 0.302 |  | 1.184(0.703-1.991) | 0.517 |  | 0.755(0.416-1.368) | 0.345 |  | 0.969(0.634-1.482) | 0.88 |  |  |
| Non-Hispanic White | ref |  | 0.855(0.653-1.118) | 0.245 |  | 1.005(0.794-1.273) | 0.966 |  | 0.998(0.718-1.387) | 0.990 |  | 1.134(0.777-1.654) | 0.51 |  |  |
| Non-Hispanic Black | ref |  | 0.807(0.571-1.141) | 0.219 |  | 0.975(0.734-1.293) | 0.855 |  | 0.806(0.622-1.043) | 0.099 |  | 0.791(0.565-1.109) | 0.17 |  |  |
| Other Race | ref |  | 0.793(0.531-1.182) | 0.247 |  | 0.994(0.683-1.445) | 0.974 |  | 0.78(0.453-1.344) | 0.362 |  | 0.968(0.571-1.641) | 0.90 |  |  |
| Education level |  |  |  |  |  |  |  |  |  |  |  |  |  |  | 0.027 |
| Less than high school | ref |  | 0.887(0.464-1.697) | 0.711 |  | 0.856(0.613-1.196) | 0.353 |  | 0.876(0.577-1.328) | 0.524 |  | 0.723(0.499-1.047) | 0.09 |  |  |
| High school | ref |  | 0.934(0.588-1.484) | 0.767 |  | 1.185(0.891-1.574) | 0.236 |  | 0.873(0.571-1.333) | 0.520 |  | 1.041(0.691-1.568) | 0.84 |  |  |
| More than high school | ref |  | 0.794(0.636-0.992) | 0.042 |  | 0.965(0.805-1.157) | 0.695 |  | 0.934(0.744-1.173) | 0.549 |  | 0.995(0.72-1.377) | 0.98 |  |  |
| Marital status |  |  |  |  |  |  |  |  |  |  |  |  |  |  | 0.735 |
| Married/living with partner | ref |  | 0.811(0.63-1.044) | 0.101 |  | 1.047(0.879-1.248) | 0.596 |  | 0.955(0.754-1.21) | 0.694 |  | 1.01(0.736-1.387) | 0.95 |  |  |
| Widowed/divorced/separated | ref |  | 0.849(0.503-1.435) | 0.532 |  | 0.862(0.594-1.25) | 0.424 |  | 0.988(0.548-1.781) | 0.968 |  | 0.929(0.555-1.554) | 0.77 |  |  |
| Never married | ref |  | 0.931(0.616-1.408) | 0.729 |  | 1.065(0.738-1.535) | 0.731 |  | 0.783(0.571-1.073) | 0.125 |  | 0.9(0.62-1.306) | 0.57 |  |  |
| Poverty-income ratio |  |  |  |  |  |  |  |  |  |  |  |  |  |  | 0.037 |
| <1.3 | ref |  | 0.711(0.497-1.016) | 0.061 |  | 1.013(0.756-1.358) | 0.928 |  | 0.79(0.491-1.272) | 0.324 |  | 0.94(0.661-1.336) | 0.72 |  |  |
| 1.3~3.5 | ref |  | 0.79(0.532-1.173) | 0.235 |  | 0.919(0.721-1.172) | 0.486 |  | 1.02(0.792-1.315) | 0.872 |  | 0.874(0.637-1.199) | 0.39 |  |  |
| >3.5 | ref |  | 0.897(0.659-1.221) | 0.482 |  | 1.073(0.799-1.442) | 0.632 |  | 0.913(0.652-1.279) | 0.589 |  | 1.056(0.665-1.676) | 0.81 |  |  |
| Not recorded | ref |  | 0.691(0.402-1.188) | 0.176 |  | 0.896(0.612-1.311) | 0.562 |  | 0.752(0.428-1.322) | 0.313 |  | 0.976(0.592-1.611) | 0.92 |  |  |
| Alcohol drinking status |  |  |  |  |  |  |  |  |  |  |  |  |  |  | 0.774 |
| Non-drinkers | ref |  | 0.591(0.27-1.291) | 0.181 |  | 0.769(0.429-1.379) | 0.369 |  | 0.723(0.317-1.646) | 0.430 |  | 0.948(0.445-2.017) | 0.89 |  |  |
| Moderate drinkers | ref |  | 1.039(0.774-1.394) | 0.795 |  | 1.11(0.844-1.461) | 0.446 |  | 1.027(0.716-1.472) | 0.883 |  | 0.949(0.657-1.371) | 0.77 |  |  |
| Heavy drinkers | ref |  | 0.633(0.468-0.858) | 0.004 |  | 0.904(0.72-1.134) | 0.374 |  | 0.814(0.557-1.19) | 0.281 |  | 0.958(0.679-1.351) | 0.80 |  |  |
| Not recorded | ref |  | 1.083(0.66-1.778) | 0.746 |  | 1.105(0.774-1.578) | 0.575 |  | 1.017(0.649-1.595) | 0.939 |  | 0.928(0.675-1.275) | 0.64 |  |  |
| Smoking status |  |  |  |  |  |  |  |  |  |  |  |  |  |  | 0.459 |
| Never | ref |  | 0.827(0.663-1.031) | 0.089 |  | 1.045(0.835-1.307) | 0.694 |  | 1.014(0.791-1.298) | 0.912 |  | 0.891(0.687-1.155) | 0.373 |  |  |
| Former | ref |  | 0.801(0.536-1.196) | 0.270 |  | 1.03(0.769-1.378) | 0.841 |  | 0.709(0.477-1.054) | 0.087 |  | 1.165(0.719-1.887) | 0.527 |  |  |
| Now | ref |  | 1.022(0.638-1.636) | 0.928 |  | 0.83(0.613-1.124) | 0.222 |  | 0.798(0.451-1.413) | 0.430 |  | 0.898(0.525-1.536) | 0.687 |  |  |
| Not recorded | ref |  |  |  |  |  |  |  |  |  |  |  |  |  |  |
| BMI |  |  |  |  |  |  |  |  |  |  |  |  |  |  | 0.131 |
| < =25kg/m2 | ref |  | 0.778(0.54-1.122) | 0.174 |  | 0.849(0.644-1.12) | 0.239 |  | 0.974(0.509-1.865) | 0.935 |  | 1.059(0.54-2.076) | 0.87 |  |  |
| 25-30kg/m2 | ref |  | 0.65(0.432-0.978) | 0.039 |  | 1.062(0.815-1.384) | 0.648 |  | 0.825(0.619-1.099) | 0.183 |  | 0.936(0.685-1.278) | 0.67 |  |  |
| > 30kg/m2 | ref |  | 0.98(0.744-1.291) | 0.882 |  | 1.025(0.811-1.295) | 0.835 |  | 0.943(0.724-1.228) | 0.654 |  | 0.96(0.723-1.274) | 0.77 |  |  |
| Not recorded | ref |  | 0.8(0.211-3.034) | 0.737 |  | 0.293(0.082-1.052) | 0.059 |  | 0.486(0.158-1.494) | 0.201 |  | 0.285(0.057-1.435) | 0.12 |  |  |
| Sedentary behavior |  |  |  |  |  |  |  |  |  |  |  |  |  |  | 0.501 |
| <=7.5h | ref |  | 0.846(0.69-1.037) | 0.104 |  | 1.049(0.886-1.243) | 0.571 |  | 1.001(0.832-1.205) | 0.991 |  | 1.023(0.779-1.344) | 0.864 |  |  |
| >7.5h | ref |  | 0.792(0.574-1.091) | 0.149 |  | 0.929(0.73-1.182) | 0.541 |  | 0.782(0.516-1.185) | 0.239 |  | 0.889(0.62-1.276) | 0.52 |  |  |
| Not recorded | ref |  | 3.762(0.115-123.146) | 0.446 |  | 0.2(0.012-3.461) | 0.260 |  | 3.961(0.292-53.765) | 0.291 |  | 0.392(0.014-10.792) | 0.57 |  |  |
| Diabetes |  |  |  |  |  |  |  |  |  |  |  |  |  |  | 0.419 |
| No | ref |  | 0.805(0.668-0.971) | 0.024 |  | 1.027(0.87-1.212) | 0.749 |  | 0.94(0.71-1.245) | 0.658 |  | 0.991(0.778-1.262) | 0.94 |  |  |
| Yes | ref |  | 1.032(0.611-1.742) | 0.904 |  | 0.81(0.547-1.201) | 0.286 |  | 0.798(0.498-1.28) | 0.340 |  | 0.919(0.645-1.31) | 0.63 |  |  |
| Prediabetes |  |  |  |  |  |  |  |  |  |  |  |  |  |  | 0.133 |
| No | ref |  | 0.779(0.636-0.956) | 0.018 |  | 0.946(0.796-1.125) | 0.524 |  | 0.917(0.75-1.123) | 0.393 |  | 0.858(0.668-1.102) | 0.23 |  |  |
| Yes | ref |  | 0.895(0.639-1.255) | 0.512 |  | 1.106(0.849-1.442) | 0.446 |  | 0.923(0.624-1.364) | 0.680 |  | 1.17(0.846-1.619) | 0.33 |  |  |
